# Recommendation on screening for chlamydia and gonorrhea in primary care for individuals not known to be at high risk

**DOI:** 10.1503/cmaj.201967

**Published:** 2021-04-19

**Authors:** Ainsley Moore, Gregory Traversy, Donna L. Reynolds, John J. Riva, Guylène Thériault, Brenda J. Wilson, Melissa Subnath, Brett D. Thombs

**Affiliations:** Department of Family Medicine (Moore), McMaster University, Hamilton, Ont.; Public Health Agency of Canada (Subnath, Traversy), Ottawa, Ont.; Department of Family and Community Medicine (Reynolds), University of Toronto, Toronto, Ont.; Department of Family Medicine (Riva), McMaster University, Hamilton, Ont.; Faculty of Medicine (Thériault) McGill University, Montréal, Que.; Division of Community Health and Humanities (Wilson), Memorial University, NFLD; Lady Davis Institute and Department of Psychiatry (Thombs), Jewish General Hospital and McGill University, Montréal, Que.

**KEY POINTS**
- *Chlamydia trachomatis* and *Neisseria gonorrhoeae* are the most commonly reported sexually transmitted bacterial infections in Canada and can cause complications including pelvic inflammatory disease, chronic pelvic pain, ectopic pregnancy and infertility.
- Opportunistic offering of screening for chlamydia in primary care may reduce pelvic inflammatory disease in females, although the evidence is uncertain.
- Most patients likely prioritize the potential benefits of screening over harms; a small proportion of those eligible for screening may experience psychosocial harms of embarrassment, anxiety or stigma.
- The Canadian Task Force on Preventive Health Care recommends screening of sexually active individuals younger than 30 years for chlamydia and gonorrhea annually at primary care visits, as feasible (conditional recommendation; very low-certainty evidence).

Key messages for the public

- Chlamydia and gonorrhea are common sexually transmitted infections that are treatable with antibiotics.
- If you are younger than age 30 and sexually active, your health care provider may offer screening for chlamydia and gonorrhea every year, even if you do not have symptoms.
- Screening is done to find and treat infections in people who aren’t showing symptoms, which may reduce complications like pelvic inflammatory disease. These infections are often without symptoms.
- Talk to your doctor about screening more than once per year if you think you are at increased risk of infection (e.g., you have had a sexually transmitted infection, you have had unprotected sex, sex with multiple partners, or another reason).
- Chlamydia and gonorrhea infections are automatically reported to local public health units. This is to help with treatment for people who test positive and also with confidentially notifying sexual partners so that they may be tested and treated as required.

In Canada, *Chlamydia trachomatis* (chlamydia) and *Neisseria gonorrhoeae* (gonorrhea) are the most commonly reported sexually transmitted bacterial infections (STIs),^1^,^2^ with reported cases in the population increasing annually since 2000.^3^ In 2018, reported cases were highest in people aged 15–29 years, with rates of 1.0%–1.9% for chlamydia and 0.2%–0.3% for gonorrhea in this population,^4^ while rates among individuals older than 30 years were less than 0.5% for chlamydia and less than 0.2% for gonorrhea. 4 Many infected individuals, however, are asymptomatic or do not seek care, and are not included in reported cases.^3^ Taking into account underreporting, the true prevalence of chlamydia in people aged 15 to 29 years may be as high as 5%–7%.^3^–^6^

Consequences of untreated chlamydia in females can include cervicitis in 10%–20%,^7^ pelvic inflammatory disease in 10%–16%,^8^,^9^ infertility in up to 5%,^10^ chronic pelvic pain in 3%–8%,^10^,^11^ and ectopic pregnancy in up to 2%.^10^ Consequences of untreated gonorrhea may include rates of pelvic inflammatory disease that exceed those for chlamydia.^12^ In males, chlamydia may be associated with epididymitis in up to 7%, with or without orchitis,^6^,^13^ and, very rarely, infertility.^14^ Consequences of chlamydia affecting both sexes include urethritis in 4% of females and up to 3% of males;^15^ pharyngitis; proctitis; reactive arthritis lasting longer than 6 months in 1%–4% (when considering both chlamydia and gonorrhea);^16^,^17^ and disseminated gonococcal infection in less than 1%, which can rarely lead to sepsis, meningitis, endocarditis or osteomyelitis.^18^

Screening sexually active individuals for chlamydia and gonorrhea could reduce clinical complications and transmission, but should be done only if benefits from screening exceed harms^19^,^20^ and resource use is justifiable.

In Canada, STI screening is most commonly offered opportunistically by clinicians in a variety of primary care settings (e.g., family practice, sexual health clinics, school health centres) during visits that may or may not be for sexual health–related concerns.^21^ Opportunistic screening is distinct from a systematic population screening program, in which invitations for screening are sent to all eligible participants, monitored for uptake and evaluated through a centralized program, usually at the provincial level.

National guidance from the Public Health Agency of Canada currently recommends screening for chlamydia in pregnant women, annual screening for sexually active individuals younger than 25 years, and targeted screening of at-risk individuals older than 25 years (e.g., those with previous STIs, sex trade workers).^21^–^23^ This guidance is not based on formal systematic reviews and does not include recommendations for gonorrhea screening.

The last guideline on chlamydia screening from the Canadian Task Force on the Periodic Health Examination (now Task Force on Preventive Health Care) was published in 1996;^24^ it also did not include recommendations on gonorrhea. Therefore, the task force identified a need for an updated Canadian guideline that considers current evidence on the potential harms, benefits, and patient values and preferences of screening for chlamydia and gonorrhea.

## Scope

This guideline is intended for clinicians in primary care settings who are positioned to offer opportunistic screening for chlamydia and gonorrhea directly to sexually active individuals who are not specifically seeking care for a possible STI and are not known to belong to a high-risk group. Readers should refer to relevant national, provincial or local guidance for the screening of individuals known to have specific high-risk behaviours (these will vary by jurisdiction, but may include having multiple sexual partners, previous STIs or sex without condoms^22^,^25^,^26^); testing of individuals seeking care for symptoms; pregnant individuals; and for selection of appropriate antibiotic treatment, partner notification, retesting and forensic testing strategies. The terms “male” and “female,” when used, refer to sex (i.e., biological attributes, particularly the reproductive or sexual anatomy at birth), unless otherwise indicated.

## Recommendation

We recommend opportunistic screening of sexually active individuals younger than 30 years who are not known to belong to a high-risk group, annually, for chlamydia and gonorrhea at primary care visits, using a self-or clinician-collected sample (conditional recommendation; very low-certainty evidence).

A summary of the recommendation is provided in Box 1.

Box 1: **Summary of recommendation for clinicians, policy-makers and patients**

We recommend opportunistic screening of sexually active individuals younger than 30 years who are not known to belong to a high-risk group, annually, for chlamydia and gonorrhea at primary care visits, using a self-or clinician-collected sample (conditional recommendation; very low-certainty evidence).

Providers should refer to relevant national, provincial or local guidance for screening of individuals known to belong to specific high-risk groups.

In the systematic review conducted for this guideline, all relevant studies (9 randomized controlled trials [RCTs],^5^,^6^,^8^,^27^–^32^ 1 controlled clinical trial^33^ and 2 retrospective cohort studies^34^,^35^) on the potential benefits of chlamydia screening provided indirect evidence (i.e., low applicability) on how and to whom screening would be offered in Canadian primary care. For example, 4 RCTs offered screening (regardless of uptake) by mailed invitation or through public education and screening encouragement^6^,^29^ rather than via direct discussion, and 1 cluster RCT provided clinic-level interventions (packages to help encourage clinicians to offer screening)^5^ rather than direct clinician engagement, yielding low clinician participation and offers of screening. Three trials evaluated only those who accepted screening (acceptors of screening),^8^,^28^,^33^ and 1 trial evaluated an offer to screen among those preselected owing to an interest in screening (offer to screen, preselected),^31^ which is indirect to the varied screening interest and acceptance among Canadian primary care patients. No studies on the effects of screening for gonorrhea for any outcomes of interest were identified in general-risk populations.

Eleven studies were identified on harms of screening for chlamydia. One RCT reported on adverse antibiotic events,^5^ and 10 uncontrolled cohort studies reported on psychosocial harms.^20^,^36^–^44^ No studies examined harms of screening for gonorrhea.

Fourteen studies examined values and preferences of screening for chlamydia or gonorrhea: 4 studies measured health state utility values (a measure of preference for being in a particular state of health^45^) for several of the benefit outcomes of screening for chlamydia and gonorrhea^11^,^46^–^48^ infection, and 10 (survey and qualitative) studies considered the relative importance of benefits to harms of chlamydia screening.^49^–^58^

### Benefits of screening

#### Offer to screen, regardless of uptake

Meta-analysis of 2 RCTs (*n* = 141 362) found very low-certainty evidence for little to no difference in pelvic inflammatory disease rate among females aged 16–29 years, over 1 to 3 years using an annual offer of chlamydia screening via self-collected vaginal samples (0.3 more in 1000 [95% confidence interval (CI) 7.6 fewer to 11 more]).^5^,^6^

One RCT (*n* = 15 459) found very uncertain effects on infertility and very low-certainty evidence for little to no difference in ectopic pregnancy rates for females aged 21 to 24 years, over 9 years from a single offer of chlamydia screening via self-collected vaginal samples (0.2 more in 1000 [95% CI 2.2 fewer to 3.9 more]).^6^

Meta-analysis of 3 RCTs (*n* = 41 709) found low-certainty evidence for little to no difference in chlamydia transmission for individuals aged 15–29 years, over 1 to 3 years from a single offer of chlamydia screening via self-collected vaginal^5^,^27^ or urine samples^24^,^26^ (5.4 fewer per 1000 [95% CI 21.0 fewer to 12.6 more]).^27^,^29^

#### Offer to screen, preselected individuals interested in screening

One RCT (*n* = 2607) — among females aged 18–34 years (81% younger than 24 yr) preselected based on completing a prescreening questionnaire on chlamydia risk and then accepting an offer of a primary care appointment (suggesting an interest in being screened) — found low-certainty evidence that offering a single chlamydia screening via clinician-collected cervical swabs may reduce pelvic inflammatory disease (15.4 fewer per 1000 [95% CI 3.0 to 21.3 fewer], number needed to screen 65 [95% CI 47 to 333]).^31^

#### Acceptors of screening

Two RCTs and 1 controlled clinical trial (*n* = 30 652) found low-certainty evidence that females aged 15–29 years who complete a single chlamydia screen over 12–18 months via self-collected vaginal^8^,^28^ or urine samples^33^ may have a reduced risk for pelvic inflammatory disease over 1 year (5.7 fewer per 1000 [95% CI 10.8 fewer to 1.1 more]).^8^,^28^,^33^

### Harms of screening

One RCT (*n* = 37 543 tested; *n* = 4574 patients who received a diagnosis of chlamydia; number treated not reported) reported no adverse events from antibiotic treatment for chlamydia (very low-certainty evidence).^5^ Cohort studies^20^,^36^–^44^ reported on a variety of psychosocial harms of screening that were synthesized narratively; low- or very low-certainty evidence indicated that undergoing screening may lead to feelings of stigmatization (e.g., guilt, embarrassment, social disapproval) or anxiety about future infertility, sexuality or risk of infection in a small to moderate proportion of individuals (50–400 per 1000 individuals screened). The number of individuals affected in the entire eligible screening population is likely smaller. The exact duration and severity of these symptoms is unknown.

### Patient values and preferences

Considering benefits relative to harms, surveys and qualitative studies found that individuals considering screening (*n* = 777)^49^–^55^ or undergoing screening (*n* = 77)^56^–^58^ placed greater relative importance on potential reproductive health and decreased transmission benefits than on anxiety or stigma of screening (very low-certainty evidence). No studies considered patient values related to adverse events from medication.

Similarly, the patient engagement study that the task force conducted for this guideline (described in Methods) showed that patients likely prioritize potential benefits of screening (all rated critical or important) over harms (all rated important) and have a strong preference to be screened; this was the case even when participants were presented with the evidence and its uncertainty.^59^,^60^

Considering the relative prioritization of different screening benefits, studies reporting health state utilities found that while utility values are similar across benefit outcomes,^11^,^46^–^48^ when considering durations of the health states, the avoidance of infertility and chronic pelvic pain may be more important to females than ectopic pregnancy, pelvic inflammatory disease or cervicitis (low-to-moderate certainty).^61^

### Resource use

The task force did not conduct a systematic review on resource use or cost-effectiveness. In the judgment of the task force, the recommendation to screen all eligible patients at opportunistic visits could involve additional (moderate) costs, largely because of additional clinician time at these encounters and testing costs.

Cost-effectiveness estimates based on opportunistic screening scenarios suggest that high versus low rates of screening may improve cost-effectiveness,^62^ and that screening may be cost-effective in Canada provided that the probability of chlamydia progressing to pelvic inflammatory disease is at least 10%,^63^ although this is of very low certainty.

### Feasibility, acceptability, cost and equity

Screening is currently a part of primary care practice and therefore judged to be feasible and likely acceptable to primary care practitioners and patients. Notably, 1 included RCT showed that patients accepted screening 80% of the time that it was offered^64^ (although the overall screening rate in this trial was low [24%] because of lack of offer).^5^

The task force anticipates that public health and other policymakers will find the recommendation to screen acceptable, given the number of people affected, increasing incidence of chlamydia and gonorrhea infection,^4^ and availability of effective treatment.

In the judgment of the task force, the recommendation would likely improve health equity by normalizing screening as routine for sexually active individuals and thereby reducing important barriers to screening, such as fear of disapproval or discrimination and feelings of stigmatization.^65^ Additionally, because females carry most of the burden of the clinical consequences of infection, screening of males (a reservoir of infection for females) may improve health equity for females.

### Rationale

#### Benefits

The indirectness (low applicability) of available evidence to inform opportunistic screening in Canada represents a major source of uncertainty, in addition to the very uncertain or lack of evidence for some outcomes of interest for chlamydia screening and for all outcomes of interest for gonorrhea screening. All evidence on benefits was of low or very low certainty, largely because of concerns about indirectness as well as imprecision (Appendix 1, available at www.cmaj.ca/lookup/doi/10.1503/cmaj.201967/tab-related-content). Pelvic inflammatory disease may be reduced for those accepting and undergoing chlamydia screening^8^,^28^,^33^ and for those interested in being screened who are offered it (low certainty)^31^ (Table 1). Very uncertain evidence found little to no difference in pelvic inflammatory disease when chlamydia screening was offered, via mailed invitation or clinic-level packages that encouraged screening. The task force judged that the true benefit of chlamydia screening when offered directly by Canadian primary care practitioners, who are positioned to identify those eligible and to offer screening opportunistically, would likely lie within this observed range of screening effectiveness (Table 1).

**Table 1:** Effects of screening for chlamydia on pelvic inflammatory disease among general-risk individuals

Outcome	Approach	No. of studies and design	Follow-up period, mo	Rate in unscreened individuals	Rate in screened individuals (95% CI)	Absolute difference (95% CI)	Certainty of evidence
Pelvic inflammatory disease	Offer of screening,* all eligible	2 RCTs^5^,^6^ *n* = 141 362	12–36	27.0 per 1000†	27.3 per 1000 (19.4 to 38.0)	0.30 more in 1000 (7.60 fewer to 11.0 more)	⊕⊖⊖⊖ VERY LOW‡,§
	Offer of screening,* selected individuals	1 RCT^31^ *n* = 2607	12	27.0 per 1000†	11.6 per 1000 (5.70 to 24.0)	15.4 fewer per 1000 (3.00 to 21.30 fewer)	⊕⊕⊖⊖ LOW§,¶
	Acceptors of screening	2 RCTs, 1 CCT^8^,^28^,^33^ *n* = 30 652	12–18	27.0 per 1000†	21.3 per 1000 (16.2 to 28.1)	5.70 fewer per 1000 (10.8 fewer to 1.10 more)	⊕⊕⊖⊖ LOW‡,§

Note: CCT = controlled clinical trial, CI = confidence interval, CT = *Chlamydia trachomatis*, PID = pelvic inflammatory disease, RCT = randomized controlled trial.

* These analyses represent results of studies that examined the effect of offering chlamydia or gonorrhea screening to all eligible individuals, regardless of level of uptake. One study used an offer of screening approach in a preselected population of individuals interested in screening (offer of screening, selected individuals).

† The effects without screening assumed that about 6% of the female population would have chlamydia (general-risk prevalence). For the outcome of PID, it was assumed that about 13% of females with chlamydia would develop PID (0.78% of the total population), and that about 25%–30% of all-cause PID is attributed to chlamydia (all-cause PID = 3.5 times PID from CT); 0.78% × 3.5 = 2.7% prevalence of PID owing to chlamydia in the unscreened group.

‡ Serious concerns about indirectness.

§ Serious concerns about imprecision.

¶ Serious concerns about risk of bias.

The recommendation to screen individuals younger than 30 years is based on the fact that almost all of the underlying evidence comes from studies of individuals in this age group. Further, the rates of chlamydia and gonorrhea are increasing among those aged 25–29 years in Canada, with rates and total cases similar to those aged 15–19 years.^4^,^60^ Conversely, rates of chlamydia for those aged 30–39 years are less than 50% of those for individuals 15–19 and 24–29 years, and less than 25% of those for individuals aged 20–24 years.^4^ Similarly, rates in those aged 40–59 years are less than 25% of those in individuals aged 30–39 years.^4^

Considering the properties of sexual networks, this recommendation to screen sexually active males as well is intended to reduce chlamydia and gonorrhea infection and its negative consequences in females, through their role in the transmission of these infections (although there were no available studies informing this rationale).

The task force made the recommendation to screen for gonorrhea as well (despite the lack of available evidence) given that many gonorrhea cases are asymptomatic;^23^,^66^ up to 40% of those with gonorrhea may have concurrent chlamydia;^67^–^69^ and current Canadian clinical and laboratory practice is to combine testing for gonorrhea with chlamydia using a single sample (most commercial nucleic acid amplification test [NAAT] assays test for both organisms simultaneously with a single specimen^25^). The incremental costs of screening for both chlamydia and gonorrhea (versus, for example, chlamydia alone) is uncertain but likely minimal, as some provincial schedules already include NAAT for chlamydia and gonorrhea together under a single price.^70^

#### Harms

The task force placed a lower priority on the very uncertain evidence of no serious adverse effects of antibiotic treatment for chlamydia and gonorrhea and uncertain evidence for psychosocial harms of screening (anxiety, shame and stigma) that are likely to be experienced by a small proportion of those eligible for screening.

The task force judged that the potential benefits of screening for chlamydia and gonorrhea to reduce pelvic inflammatory disease in females, albeit very uncertain, outweigh possible harms. Evidence suggests that most Canadian patients also prioritize the benefits over the harms of screening for chlamydia and gonorrhea, even when provided with the evidence and its uncertainty. 59,60 Therefore, considering the balance of benefits and harms as well as evidence uncertainty, the task force provides a conditional recommendation in favour of opportunistic screening for chlamydia and gonorrhea in primary care for individuals younger than 30 years.

## Methods

The Canadian Task Force on Preventive Health Care is an independent panel of clinicians and methodologists that makes recommendations on primary and secondary prevention in primary care (www.canadiantaskforce.ca). A working group of 6 task force members developed this recommendation, with scientific support from the science team at the Public Health Agency of Canada.

The working group established the research questions and the analytical framework for the systematic review (Appendix 2, available at www.cmaj.ca/lookup/doi/10.1503/cmaj.201967/tab-related-content). The Evidence Review and Synthesis Centre at the University of Alberta (Edmonton) conducted the systematic review on which the recommendation is based, which evaluated the effectiveness of chlamydia and gonorrhea screening and the relative importance of screening outcomes (benefits and harms) to patients.^61^

A search for eligible studies was conducted using Ovid MEDLINE, Ovid Embase, Wiley Cochrane Library, CINAHL via EBSCOhost, and Ovid PsycINFO from database inception to Jan. 26, 2020, with supplemental searches for grey literature. For the relative importance of potential benefit outcomes, a 2013 review on health state utility values was also updated.^71^ The systematic review was registered (PROSPERO CRD42018100733), and a protocol for the review was published.^13^

In the systematic review, potential benefits of screening examined included reduced pelvic inflammatory disease, ectopic pregnancies, cervicitis and chronic pelvic pain in females; and for both females and males, reduced infertility and transmission (prevalence). Potential harms examined included serious adverse antibiotic reactions and negative psychosocial implications of screening (e.g., anxiety, sexual relationship distress including partner violence, stigmatization, blame). Thresholds for minimally important differences in benefit outcomes were determined before the task force examined the results. Details can be found in the systematic review.^13^,^61^

The task force used the Grading of Recommendations, Assessment, Development and Evaluation (GRADE) approach to determine the certainty of evidence and strength of recommendation (Box 2). See Appendix 3, available at www.cmaj.ca/lookup/doi/10.1503/cmaj.201967/tab-related-content. The entire task force approved the recommendations.

Box 2: **Grading of recommendations**

Recommendations are graded according to the Grading of Recommendations Assessment, Development and Evaluation system (GRADE).^72^ Whether a recommendation is strong or conditional is based on considerations such as certainty in the effects of an intervention, including magnitude, as well as estimates of how patients value and prioritize outcomes, variability of these estimates and wise use of resources.

**Strong recommendations**

- Strong recommendations are those for which the Canadian Task Force on Preventive Health Care is confident that the desirable effects of an intervention outweigh its undesirable effects (strong recommendation for an intervention) or that the undesirable effects of an intervention outweigh its desirable effects (strong recommendation against an intervention). A strong recommendation implies that most people will be best served by the recommended course of action.
- Strong recommendations are typically based on high-certainty evidence (i.e., high confidence in the estimate of the effect of an intervention). Strong recommendations may recommend in favour of an intervention (when there is high confidence of net benefit) or against an intervention (when there is high confidence of net harm). However, there are circumstances in which a strong recommendation may be considered based on low- or very low-certainty evidence, or when there is absence of evidence.^73^
- When there is an absence of evidence to provide confidence that there is benefit from implementing a new prevention service or when a conclusion of possible benefit requires a high level of speculation on linkages of uncertain evidence, but there is high certainty that some patients would be harmed or scarce health care resources expended, the task force may make a strong recommendation against service implementation.^74^ This is consistent with the GRADE approach, in which strong recommendations are sometimes made with low-certainty evidence combined with high certainty of harm or resource implications, and with the value that the task force places on using scarce primary care resources wisely.^74^

**Conditional recommendations**

- Conditional recommendations are those for which the desirable effects probably outweigh the undesirable effects (conditional recommendation in favour of an intervention) or undesirable effects probably outweigh the desirable effects (conditional recommendation against an intervention) but appreciable uncertainty exists. Conditional recommendations are made when the certainty of evidence is lower, when the margin between desirable and undesirable consequences is small and the balance depends on patient values and preferences, or when there is high variability in the values and preferences of patients. Conditional recommendations may also be applied when the balance of cost and benefits is ambiguous, key stakeholders differ about the acceptability or feasibility of the implementation, or the effects on health equity are unclear.
- In certain cases where a conditional recommendation for an intervention is made, clinicians are encouraged to engage in shared decision-making, to recognize that different choices will be appropriate for individual patients, and to help each person arrive at a management decision consistent with their values and preferences.

Evidence is graded as high-, moderate-, low- or very low-certainty, based on how confident the task force is that the estimates of effect are correct.

More information about the task force’s methods are available at the task force website (https://canadiantaskforce.ca/methods/).

### Patient engagement

The Knowledge Translation team at St. Michael’s Hospital (Toronto) engaged members of the public from across Canada, recruited via advertisements on public websites (i.e., Craigslist and Kijiji), on behalf of the task force at 2 stages of guideline development. As a first step, 16 sexually active participants (9 identified as female, and 7 identified as male) aged 24–38 years rated the importance of screening outcomes via online survey and participated in a focus group to share their rationale for their ratings and discuss factors that affect the perceived importance of various outcomes.^59^,^75^ Outcomes considered critical and important to decision-making by both participants and task force members were included in the systematic review. After the evidence review was completed, 17 different sexually active participants aged 24–38 years (13 participants identified as male, 3 identified as female, and 1 identified as non-binary) rated the importance of screening outcomes via online survey, this time after being provided with a summary of systematic review results.^60^

Knowledge translation tools to support recommendation implementation, informed by feedback from clinicians and patients, were developed (available at https://canadiantaskforce.ca/tools-resources/chlamydia-and-gonorrhea/).

### External and content expert review

The protocol,^13^ systematic review^61^ and draft guideline were each reviewed by stakeholders, peer reviewers, and clinical and content experts (see Acknowledgements). Clinical and content experts served as advisors to the working group; they participated in working group meetings and reviewed documents for accuracy, but did not have input into or vote on the direction or strength of recommendations.

### Management of competing interests

The task force follows Guidelines International Network principles for disclosures of interests and management of competing interests.^76^,^77^ The task force’s oversight committee for evaluating and adjudicating competing interests consists of the task force chair and vice-chair and the director of the Global Health and Guidelines Division of the Public Health Agency of Canada.^77^

Funding for the task force is provided by the Public Health Agency of Canada. The task force did not consider the views of the funding body in developing the guideline.

All task force members are required to disclose financial and other relevant interests annually when new topics are selected and at each in-person meeting of the task force (3 times per year). These disclosures are available on the task force website (https://canadiantaskforce.ca/about/members/). There were no conflicts of interest among task force members for this guideline.

Clinical and content experts also disclose relevant interests at the outset of their participation and annually thereafter (see Appendix 4, available at www.cmaj.ca/lookup/doi/10.1503/cmaj.201967/tab-related-content). The task force did not judge any disclosures to represent conflicts of interest that precluded participation as a clinical or content expert.

## Implementation

To implement this screening recommendation, clinicians in primary care settings are advised to identify individuals who are eligible for screening (sexually active individuals younger than 30 years) and not seeking testing for a possible STI, and to offer chlamydia and gonorrhea screening opportunistically (i.e., without requiring a separate screening visit, and not only during sexual health visits). As noted above, results from 1 RCT suggest that patient acceptance of screening is high when offered.^64^ As individuals at high risk of chlamydia and gonorrhea infection may not always readily self-identify or be easily identified by clinicians, this routine offer of screening applies to all sexually active individuals without clinician knowledge of their membership of a high-risk group. Sexually transmitted infections are associated with shame, embarrassment and substantial stigma, which could prevent patients from seeking screening and treatment.^65^,^78^ Routinely offering screening to all sexually active individuals has been suggested as one way to reduce stigma associated with STI testing.^78^ Those seeking testing or who are known to belong to a high-risk group should be managed according to relevant national, provincial or local guidance applicable to those populations. Sexual activity can be generally defined as ever having oral, vaginal or anal intercourse.

Informed consent, which is required for STI testing, is an additional implementation consideration. The main issues to address are those related to privacy, reporting of positive test results to local public health offices, and potential partner notification. Screening for sexually transmitted infection may cause embarrassment and anxiety for some patients. Offering screening requires sensitivity to stigmatization and fear of social disapproval, especially regarding gender, culture, behaviour and other vulnerabilities.

Although the optimal screening interval is unknown, an annual offer of screening may be appropriate for individuals at general risk, recognizing that encounters with primary care may occur less frequently. Most identified studies used annual screening^61^ and, in 1 study, most pelvic inflammatory disease cases occurring within 1 year were in individuals who were chlamydia negative at baseline (general risk).^8^ A false-positive result could cause harm without benefit to some individuals. This is particularly relevant when prevalence of chlamydia in the population is lower (e.g., 2%–3%), where the number of false positives from NAAT tests may be quite high (e.g., about 30%–60% at specificities of 97%–99%).

Although we did not identify evidence that would allow recommendation of specific screening strategies, acceptability and uptake of screening^79^–^81^ may be improved by minimally invasive sample collection methods, of which self-collected vaginal swabs from females and urine samples from males are the most accurate (NAAT).^82^ Clinician-collected swabs are likely acceptable and feasible during certain encounters (e.g., Pap testing).^83^ Ultimately, patient preference and the clinical scenario will likely dictate the preferred sampling method. Clinicians are reminded to consider pharyngeal and rectal swabs if clinically warranted, although we did not identify any evidence to evaluate screening using samples from these sites.

In cases of actual or suspected child abuse, clinicians are directed to their local, provincial and territorial authorities (public health offices, child protection services, pediatricians and clinical experts), for STI testing, treatment, reporting and management.

## Monitoring and evaluation

Rates of offer and uptake of screening among patients in primary care settings are a key performance measure for this guideline. Rates of reported chlamydia and gonorrhea infections represent another performance metric.

## Other guidelines

Guidelines from several groups within Canada^22^,^25^,^26^ and internationally^84^–^86^ similarly recommend that clinicians opportunistically offer screening to sexually active individuals for chlamydia. Some, but not all, also include recommendations to screen for gonorrhea (Table 2). The present recommendation extends to age 29 years (see recommendation rationale above),^4^ whereas other guidelines recommend screening to age 25 years, except for Australia, which recommends screening to age 30 years.

**Table 2:** Canadian (national and provincial) and international guidelines on screening for chlamydia and gonorrhea

Organization	Recommendation
Canadian Task Force on Preventive Health Care (current guideline, 2021)	We recommend opportunistic screening of sexually active individuals younger than 30 yr, who are not known to belong to a high-risk group, annually, for chlamydia and gonorrhea, at primary care visits, using a self- or clinician-collected sample (conditional recommendation; very low-certainty evidence).
Public Health Agency of Canada (2020)^22^	**Chlamydia** Screening for *Chlamydia trachomatis* is recommended for anyone with risk factors for infection. Screening recommendations for the detection of *C. trachomatis*: Annual screening: Age < 25 yr Gay, bisexual, and other men who have sex with men and transgender populations Targeted screening: Offer screening and repeat screening based on risk factors in those aged ≥ 25 yr
Public Health Ontario (2018)^25^	**Gonorrhea** Offer screening to asymptomatic sexually active individuals with risk factors for gonorrhea. In Ontario, risk factors for gonorrhea of particular importance among those with unprotected sexual exposure include: Sexually active women younger than 25 yr Sexually active men who have sex with men Other risk factors as listed in the Canadian Guidelines on Sexually Transmitted Infections^23^ When performing concurrent testing for gonorrhea and chlamydia, use: Urine NAAT for males Vaginal NAAT (first-line) or urine NAAT (second-line) for females when a pelvic examination is not being conducted Cervical NAAT or vaginal NAAT (first-line) or urine NAAT (second-line) for females when a pelvic examination is being conducted
Ministère de la santé et des services sociaux du Québec (2019)^26^	**Chlamydia** Screening at least annually is recommended for: Men and women aged 25 yr and younger who are sexually active with no other risk factors Men and women with new sexual partners or with more than 1 concurrent partner since their last test Individuals who have had an anonymous partner or more than 3 sexual partners in the last year Men who have sex with men Sex workers or their clients (In some cases) Individuals originating from a region where sexually transmitted and blood-borne infections are endemic **Gonorrhea** Screening at least annually is recommended for: Men (depending on region) and all women aged 25 yr and younger who are sexually active and have no other risk factors Women with new sexual partners or with more than 1 concurrent partner since their last test Individuals who have had an anonymous partner or more than 3 sexual partners in the last year Men who have sex with men Sex workers or their clients (In some cases) Individuals originating from a region where sexually transmitted and blood-borne infections are endemic
US Preventive Services Task Force (2014)^84^	**Sexually active women** The USPSTF recommends screening for chlamydia in sexually active women aged 24 years and younger and in older women who are at increased risk for infection (*Grade B recommendation*). The USPSTF recommends screening for gonorrhea in sexually active women aged 24 years and younger and in older women who are at increased risk for infection (*Grade B recommendation*). **Sexually active men** The USPSTF concludes that the current evidence is insufficient to assess the balance of benefits and harms of screening for chlamydia and gonorrhea in men (*I statement*).
Public Health England (2018)^85^	**Chlamydia** Annually or on change of sexual partner, tests should be offered to men and women younger than 25 years who have ever been sexually active. Providers should use every opportunity to offer chlamydia screening across primary care and access to services for sexual and reproductive health and genitourinary medicine.
Australasian Sexual Health Alliance (2018)^86^	**Test for chlamydia in the following situations:** Aged < 30 yr and sexually active Partner change in the last 12 months Have had an STI in past 12 months Have had a sexual partner with an STI At increased risk of complications of an STI; e.g., termination of pregnancy or intrauterine device insertion Signs or symptoms suggestive of chlamydia Patient requests a sexual health check

**Note:** NAAT = nucleic acid amplification test, STI = sexually transmitted infection, USPSTF = United States Preventive Services Task Force.

## Gaps in knowledge

We did not identify any trials that carried out screening for chlamydia or gonorrhea in a manner consistent with how screening is offered directly to patients, opportunistically, in Canadian primary care. There was also limited evidence on health outcomes of screening for chlamydia or gonorrhea in males or their specific female partners (considering sexual networks). Almost no studies included participants older than 30 years (which may be because of the low prevalence in this population). Studies comparing the impacts of different screening intervals or different screening strategies in primary care settings on health outcomes are required.

## Limitations

Thresholds for a minimally important difference were developed for benefit outcomes of interest to determine the magnitude of effect and certainty of the evidence. These thresholds were created by task force clinicians and topic experts, taking into account Canadian epidemiologic and natural history data.^13^,^61^ We did not seek patient input on these thresholds, which for some preventive interventions with substantial harms would be useful to identify patient perspectives of the threshold of benefit.

We did not assess the impact of gonorrhea treatment on antimicrobial resistance, as we focused on patient-important outcomes of screening. It is not necessarily a limitation, but the task force has made a priori choices to pool study data in a way that may differ from other groups. We did not determine the average effect of screening across all included trials because of our predetermined interest in the effects of an offer to screen, regardless of uptake, and the large variability between the trial designs in this respect. Decision-makers less concerned about this variability may have analyzed the data and judged the magnitude of effects differently, although the direction of this recommendation would not likely change.

## Conclusion

Opportunistic screening for chlamydia and gonorrhea among sexually active individuals younger than 30 years confers uncertain but potentially important benefits, particularly for prevention of pelvic inflammatory disease in females. Psychosocial harms of screening are anticipated to be relatively mild, and patients likely prioritize potential screening benefits over harms. The task force conditionally recommends in favour of screening, at primary care visits, sexually active individuals younger than 30 years who are not known to belong to a high-risk group for chlamydia and gonorrhea. Informed consent is required for screening.
